# A Model for a Correlated Random Walk Based on the Ordered Extension of Pseudopodia

**DOI:** 10.1371/journal.pcbi.1000874

**Published:** 2010-08-12

**Authors:** Peter J. M. Van Haastert

**Affiliations:** Department of Cell Biochemistry, University of Groningen, Haren, the Netherlands; Princeton University, United States of America

## Abstract

Cell migration in the absence of external cues is well described by a correlated random walk. Most single cells move by extending protrusions called pseudopodia. To deduce how cells walk, we have analyzed the formation of pseudopodia by *Dictyostelium* cells. We have observed that the formation of pseudopodia is highly ordered with two types of pseudopodia: First, de novo formation of pseudopodia at random positions on the cell body, and therefore in random directions. Second, pseudopod splitting near the tip of the current pseudopod in alternating right/left directions, leading to a persistent zig-zag trajectory. Here we analyzed the probability frequency distributions of the angles between pseudopodia and used this information to design a stochastic model for cell movement. Monte Carlo simulations show that the critical elements are the ratio of persistent splitting pseudopodia relative to random de novo pseudopodia, the Left/Right alternation, the angle between pseudopodia and the variance of this angle. Experiments confirm predictions of the model, showing reduced persistence in mutants that are defective in pseudopod splitting and in mutants with an irregular cell surface.

## Author Summary

Even in the absence of external information, many organisms do not move in purely random directions. Usually, the current direction is correlated with the direction of prior movement. This persistent random walk is the typical way that simple cells or complex organisms move. Cells with poor persistence exhibit Brownian motion with little displacement. In contrast, cells with strong persistence explore much larger areas. We have explored the principle of the persistent random walk by analyzing how *Dictyostelium* cells extend protrusions called pseudopodia. These cells can extend a new pseudopod in a random direction. However, usually cells use the current pseudopod for alternating right/left splittings, by which they move in a persistent zig-zag trajectory. A stochastic model was designed for the persistent random walk, which is based on the observed angular frequencies of pseudopod extensions. Critical elements for persistent movement are the ratio of de novo and splitting pseudopodia, and, unexpectedly, the shape of the cell. A relatively round cell moves with much more persistence than a cell with an irregular shape. These predictions of the model were confirmed by experiments that record the movement of mutant cells that are specifically defective in pseudopod splitting or have a very irregular shape.

## Introduction

Eukaryotic cells move by extending pseudopodia, which are actin-filled protrusions of the cell surface [1]. Pseudopod formation by *Dictyostelium* cells, like many other moving cells, shows a typical pseudopod cycle: upon their initiation, pseudopodia grow at a constant rate during their first ∼15 s and then stop. The next pseudopod is typically formed a few seconds later, but sometimes commences while the present pseudopod is still growing, giving rise to a cell with two pseudopodia. The fate of the pseudopod after its initial growth phase determines its role in cell movement: the pseudopod is either retracted, or is maintained by flow of the cytoplasm into the pseudopod thereby moving the cell body. The frequency, position and directions of the maintained pseudopodia form the basis of cell movement, because they determine the speed and trajectory of the cell. An important aspect of cell motility is the ability of cells to respond to directional cues with oriented movement. Gradients of chemicals give rise to chemotaxis [2]. Other directional cues that can induce oriented movement are temperature gradients (thermotaxis) or electric fields (electrotaxis) [3], [4]. These signals somehow modulate basal pseudopod extension such that, on average, cells move in the direction of the positional cues. In this respect, studies on cell movement are critical for understanding directional movement.

Cells in the absence of external cues do not move in random directions but exhibit a so-called correlated random walk [5]–[9]. This tendency to move in the same direction is called persistence, and the duration of the correlation is the persistence time. Cells with strong persistence make fewer turns, move for prolonged periods of time in the same direction, and thereby effectively penetrate into the surrounding space. Other search strategies for efficient exploration are local diffusive search and Levi walks [8], [10]. Can we understand the cell trajectory by analyzing how cells extend pseudopodia?

To obtain large data sets of extending pseudopodia we developed a computer algorithm that identifies the cell contour and its protrusions. The extending pseudopod is characterized by a vector that connects the x,y,t coordinates of the pseudopod at the beginning and end of the growth phase, respectively [11]. A picture of ordered cell movement has emerged from the analysis of ∼6000 pseudopodia that are extended by wild type and mutant cells in buffer [12]. *Dictyostelium* cells, as many other eukaryotic cells, may extend two types of pseudopodia: *de novo* at regions devoid of recent pseudopod activity, or by splitting of an existing pseudopod [12], [13]. Pseudopod splitting occurs very frequently alternating to the right and left at a relatively small angle of ∼55 degrees. Therefore, pseudopod splitting may lead to a persistent zig-zag trajectory [14]. In contrast, de novo pseudopodia are extended in all directions and do not exhibit a right/left bias, suggesting that de novo pseudopodia induce a random turn of the cells. We observed strong persistence for cells that extend many splitting pseudopodia. Conversely, mutants that extend mostly de novo pseudopodia have very short persistence time and exhibit a near Brownian random walk [12].

In this report we investigated the theory of correlated random walks in the context of the observed ordered extension of pseudopodia. The aim is to define the descriptive persistence time or average turn angle with primary experimentally-derived pseudopod properties. First we obtained detailed quantitative data on the probability frequency distributions of the size and direction of pseudopod activity. We then formulated a model that consists of five components: pseudopod size, fraction of splitting pseudopodia, alternating right/left bias, angle between pseudopodia and variance of this angle due to irregularity of cell shape. We measured the parameter values of these components for several *Dictyostelium* mutants with defects in signaling pathways or cytoskeleton functions. Subsequently, we used these observed parameters in Monte Carlo simulations of the model and compared the predicted trajectories with the observed trajectories of the mutants. The results demonstrate two critical components in these correlated random walks: the ratio of pseudopod splitting relative to de novo pseudopodia, and the shape of the cell.

## Methods

### *Dictyostelium* strains and cell recordings

The strains used are wild type AX3, *pi3k*-null strain GMP1 with a deletion of *pi3k1* and *pi3k2* genes [15], *pla2*-null with a deletion of the *plaA* gene [16], *sgc/gca*-null cells (abbreviated as *gc*-null cells) with a deletion of *gca* and *sgc* genes [17], *sgc/pla2*-null cells with a deletion of *sgc* and *pla2A* genes [18], and *ddia2*-null cells lacking the *forH* gene encoding the *Dictyostelium* homologue of formin [19]. Cells were grown in HG5 medium (contains per liter: 14.3 g oxoid peptone, 7.15 g bacto yeast extract, 1.36 g Na_2_HPO_4_⋅12H_2_O, 0.49 g KH_2_PO_4_, 10.0 g glucose), harvested in PB (10 mM KH2PO4/Na2HPO4, pH 6.5), and allowed to develop in 1 ml PB in a well of a 6-wells plate (Nunc). Movies were recorded at a rate of 1 frame per second for at least 15 minutes with an inverted light microscope (Olympus Type CK40 with 20× objective) and images were captured with a JVC CCD camera. Cell trajectories were recorded as the movement of the centroid of the cell as described [20].

### Pseudopod analysis

**Figure 1:**
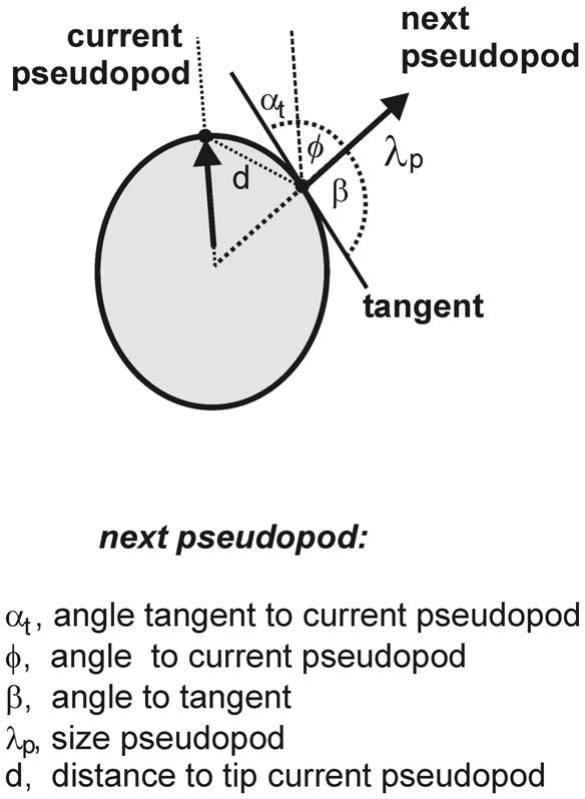
Pseudopod analysis. Movies at a rate of 1 frame per second were recorded for *Dictyostelium* cells moving on a solid support in buffer. The program Quimp3 represents the cell outline using a polygon of ∼150 nodes, and then identifies extending convex protrusions as pseudopodia, described by the x,y,t coordinates of the start and end of their growing phase. The program also calculates the tangent to the surface at the position where the pseudopod started. These data were used to calculate for each pseudopod the size *λ*, the angle *α* relative to a specific point in space, the angle *β* relative to the tangent, and the angle *ϕ* relative to the previous pseudopod.

Images were analyzed with the fully automatic pseudopod-tracking algorithm Quimp3, which is described in detail [11]. Briefly, the program uses an active contour analysis to represent the outline of the cell using ∼150 nodes [21]. Extending pseudopodia that satisfied the user-defined minimum number of adjacent convex nodes and the minimum area change were identified. The direction of each extending pseudopod was identified by the x,y and time coordinates of the central convex node of the convex area at the start and end of growth, respectively. The tangent to the surface at the node where the pseudopod started was calculated using the position of the adjacent nodes. The automated algorithm annotates each pseudopod as de novo versus splitting using the criterion that the convex area of the new pseudopod exhibits overlap with the convex area of the current pseudopod or is within a user-defined distance. The output files containing the x,y-coordinates of the start and end position of the pseudopod, the tangent of the surface and the annotation of the pseudopod were imported in Excel to calculate pseudopod size, interval, direction to gradient, direction to tangent, etc for de novo and splitting pseudopodia (see Fig. 1), as well as fraction *s* of pseudopod splitting and alternating Right/Left bias *a* (RL +LR)/total splitting; Table 1).

**Table 1:** Observed and deduced parameters of wild type and mutant cells.

Property	Symbol	Units	WT 1h	WT 3h	WT 5h	WT 7h	*gc*-null	*pla2*-null	*sgc/pla2*-null	*ddia2*-null
***Observed pseudopod*** (n cells/pseudopodia)			7/215	8/256	28/835	7/294	7/312	8/208	8/219	8/164
Pseudopod size	*λ*_p_	µm	5.0±0.2	5.3±0.2	5.2±0.2	4.7±0.2	4.6±0.4	7.7±0.5	5.3±0.7	5.6±0.4
Splitting angle	*ϕ*	degrees	62	58	55	55	54	50	54	54
SD splitting angle	*σ_ϕ_*	degrees	26.1	29.7	27.8	27.5	26.9	28.5	27.5	46.5
Alternating	*a*	-	0.74±0.02	0.74±0.06	0.77±0.04	0.82±0.06	0.67±0.05	0.68±0.05	0.75±0.08	0.75±0.03
Fraction splitting	*s*	-	0.55±0.07	0.60±0.05	0.86±0.06	0.89±0.05	0.71±0.06	0.67±0.10	0.41±0.07	0.82±0.09
***Observed dispersion*** (20 cells)										
Correlation factor	*γ_obs_*	-	0.46±0.11	0.52±0.07	0.74±0.09	0.81±0.10	0.58±0.11	0.55±0.11	0.35±0.08	0.53±0.07
Turn angle	*θ*	degrees	63	59	42	36	55	57	70	58
***Calculated dispersion***										
Monte Carlo correlation factor	*γ_MC_*	-	0.40	0.46	0.65	0.70	0.53	0.51	0.35	0.46
Equation (9) correlation factor	*γ_step_*	-	0.40	0.42	0.64	0.69	0.50	0.48	0.31	0.43

The observed pseudopod data were derived from wild type (WT) cells starved for the indicated times, or from mutants starved for 5h. Indicated are the number of cells and pseudopodia analyzed. The data for *λ*_p_ are the means and SEM for pseudopodia; the data for ϕ and *σ_ϕ_* are the means and SD for pseudopodia; the data for *a* and *s* are the means and SEM for cells. The data for the observed *γ_obs_* were obtained by least square curve fitting to Eq. 4 of the dispersion of 20 cells during 15 minutes; the error indicates the 95% confidence level of the fit; *θ* = cos^−1^ *γ*. The data for the MC simulated *γ_MC_* were obtained by simulating 100,000 trajectories using the observed pseudopod parameters, and the corresponding value of *γ* was computed as described for the experimentally observed *γ*. Finally, *γ_step_* was calculated with Eq. 9, only using pseudopod parameters.

The cell shape parameter *Ψ* was determined as follows: Using the outline of the cell with ∼150 nodes, two ellipsoids were constructed, the largest ellipse inside the cell outline and the smallest ellipse outside the cell outline. Then an intermediate ellipse was constructed by interpolation of the inner and outer ellipse. This intermediate ellipse makes several intersections with the cell outline, thereby forming areas of the cell that are outside the intermediate ellipse (with total surface area *O*), and areas of the intermediate ellipse that do not belong to the cell (with total surface area *I*; see Fig. S4). The intermediate ellipse was positioned in such a way that 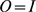 (this also implies that the surface area of the cell (T) is identical to the surface area of the intermediate ellipse). The cell shape parameter is defined as 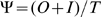; it holds that 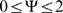. For a cell with a regular shape that approaches a smooth ellipsoid, the surface areas *O* and *I* are very small and *Ψ* approaches zero. In contrast, *O* and *I* are larger for a cell with a very irregular shape; the largest value observed among ∼600 cells was *Ψ* = 0.92.

With the exception of 5h starved cells, each database contains information from 200–300 pseudopodia, obtained from 6–10 cells, using two independent movies. For 5h starved cells, we collected a larger database containing 835 pseudopodia from 28 cells using 4 independent movies, and typical databases for each mutant. The data are presented as the means and standard deviation (SD) or standard error of the means (SEM), where n represents the number of pseudopodia or number of cells analyzed, as indicated in Table 1.

### Statistical analysis of pseudopod angles

The probability density functions of angles can not be analyzed as the common distribution on a line. Angular distributions belong to the family of circular distributions, which are constructed by wrapping the usual distribution on the real line around a circle. The data were analyzed with two circular distributions, the von Mises distribution (vMD), which matches reasonably well with the wrapped normal distribution, and the wrapped Cauchy distribution (WCD), which has fatter tails [22]. The vMD is given by
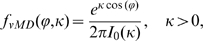
(1)where *I_0_(κ*) is the modified Bessel function of the first kind of order zero
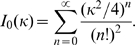
(2)The WCD is given by
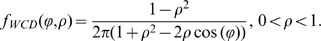
(3)


### Monte Carlo simulations

Pseudopod extension is an ordered stochastic event [12]. The position of the tip of the formed pseudopodia depends on pseudopod size *λ_p_*, splitting fraction *s*, Left/Right alternating ratio *α*, angle between split pseudopodia *ϕ* and variance of this angle *σ_ϕ_*. A Monte Carlo simulation starts with a random angle *α(1)* of the first pseudopod. For the next and all subsequent pseudopodia the simulation uses four uniformly distributed random numbers *R_i,n_* (i = 1, .., 4) to calculate *α(n)*, the angle of the n^th^ pseudopod: 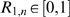 with the decision to split if *R_1,n _*<*s*; 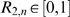 with the decision for alternating splitting if *R_2,n_* <*a*; 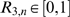 for direction of split after de novo with decision right if *R_3,n_* <0.5; and 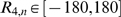 for the direction of the de novo pseudopod. These probabilities result in a projected angle of extension in degrees. Finally, the actual pseudopod direction is drawn from a wrapped von Mises distribution with this projected angle as mean and *σ_ϕ_^2^* as variance (*κ* = 1/*σ_ϕ_^2^*; variance converted to radians). The obtained *α(n)* and the pseudopod size *λ_p_* are used to calculate the x,y coordinates of the tip of the pseudopod, followed by a next round of four random numbers to calculate *α(n+1)*. In the simulations reported here we did not include stochastic variation in pseudopod length and pseudopod frequency, since we observed that they had only minor effects on the trajectories over several cell lengths.

Please note that in the simulations the direction of the simulated de novo pseudopodia is random; consequently, a small fraction of de novo pseudopodia are in the same direction of the previous pseudopod, which would be recognized in experiments as splitting pseudopodia. Conversely, a small fraction of the simulated splitting pseudopodia have angles much larger than 55 degrees and would be recognized in experiments as de novo pseudopodia. From the geometry of the cell, we estimate that the number of simulated de novo in the current pseudopod and the number of splitting pseudopodia outside the current pseudopod are approximately the same, suggesting that the simulations represent the observed ratio of splitting and de novo pseudopodia.

## Results

### Pseudopod extensions

**Figure 2:**
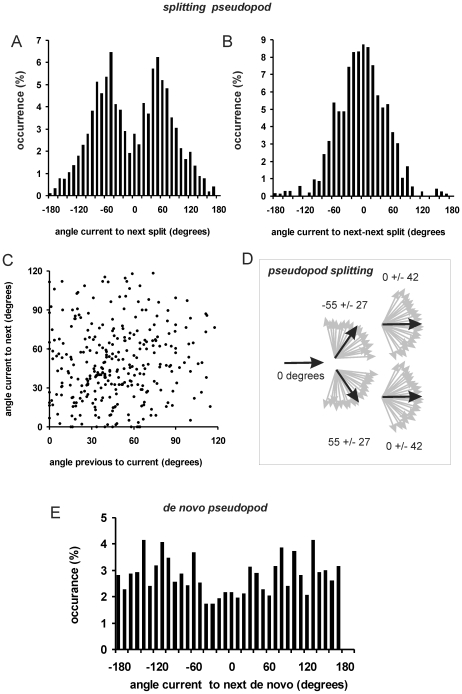
Pseudopod angles. **A**. Pseudopod extension was analyzed for the angle *ϕ*_1,2_ between current and next splitting pseudopod, yielding a bimodal von Mises distribution with mean *ϕ* = +/−55 degrees and SD = 28 degrees with 736 pseudopodia. **B**. The angle *ϕ*_1,3_ between current and next-next pseudopod exhibit a single distribution with mean *ϕ* = 2 degrees and SD = 42 degrees with 736 pseudopodia. Panel **C** shows that the angle between current and next pseudopod does not depend on the angle between current and previous pseudopod. **D**. Schematic of the alternating extension of Right/Left pseudopod splitting. **E**. The angle between current pseudopod and the next de novo pseudopod, demonstrating a nearly uniform distribution with 206 pseudopodia. Data in this figure were obtained from 3, 5 and 7h staved cells (see also Table 1).

The angles between pseudopodia were analyzed in detail and the results are presented in Fig. 2. For splitting pseudopodia, the angle between the current and next pseudopod (*ϕ*_1,2_) has a clear bimodal distribution (Fig. 2A). A probability density function (PDF) of angles belongs to the family of circular or wrapped distributions. The data reported in this study were all fitted well by a von Mises distribution (vMD), which is the circular analog of the normal distribution. The wrapped Cauchy distribution has fatter tails and provided a poorer fit of the data (data not shown). The bimodal vMD presented in Fig. 2A is symmetric, yielding two means (*ϕ*_1,2_ = +/−55) that have the same variance *κ* = 1/*σ_ϕ_^2^*; *σ_ϕ_*_1,2_ = 28 degrees). Figure 2B shows the PDF of the angle between the current and next-next pseudopod (*ϕ*_1,3_), which is best described by a single vMD with a mean of *ϕ*_1,3_ = 2 degrees and *σ_ϕ_*_1,3_ = 42 degrees. Figure 2C reveals that there is no significant correlation between the magnitude of angles between first/second pseudopod and the magnitude of the angles between second/third pseudopod (thus e.g. splitting at a larger angle is not followed by a split at a smaller angle). The extension of splitting pseudopodia is summarized in Fig. 2D, and is based on the previous observation that a pseudopod split to the right is frequently followed by a split to the left and visa versa [12]. Thus the next pseudopod is extended at an angle of ∼55 degrees to the right or left relative to the current pseudopod, and the next-next pseudopod is extended in roughly the same direction as the current pseudopod.

The angle between a de novo pseudopod and the previous pseudopod shows a very broad distribution (Fig. 2E). Nearly all angles between −180 and +180 are well represented with a somewhat lower abundance of angles around 0 degrees. This suggests that a de novo pseudopod can be extended in any direction, but with slightly lower probability of the direction of the current pseudopod.

### Trajectories of wild type and mutant cells

**Figure 3:**
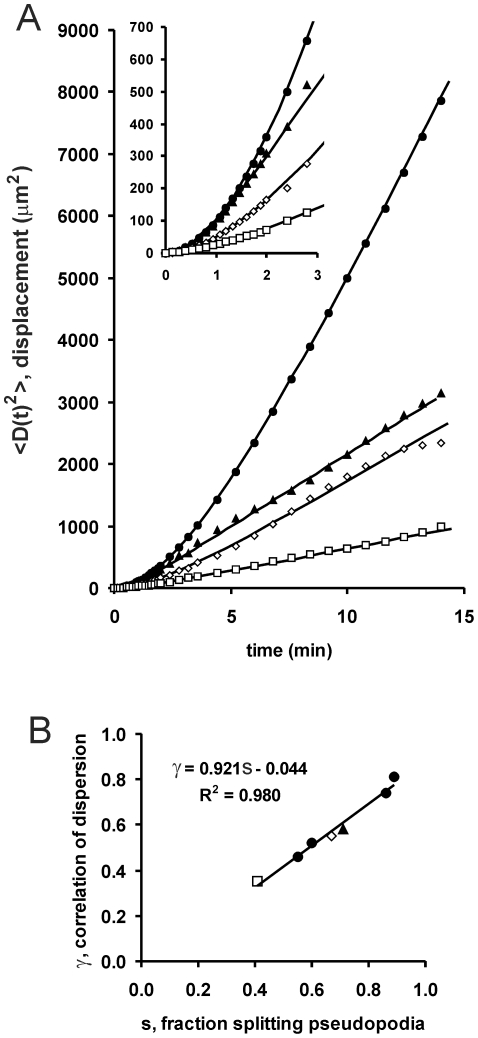
Dispersion. The trajectories of wild type and mutant cells (see Fig. S1) were recorded as described in the method section. **A**. The mean square displacement was determined for ∼20 cells (symbols). The data were fitted according Eq. 4 (lines) yielding the correlation factor of dispersion *γ*_obs_ as indicated in Table 1. **B**. The correlation factor of dispersion is plotted as a function of the fraction s of splitting pseudopodia, which was determined from the same movies. Symbols are closed circle for wild type (5 h starved in panel A; 1, 3, 5 and 7h starved in panel B); closed triangle for *sgc/gca*-null cells, diamond for *pla2*-null cells; square for *sgc/pla2*-null cells.

To investigate the consequence of the observed ordered extension of pseudopodia for cell movement on a coarse time scale for many pseudopodia we recorded the movement of *Dictyostelium* cells during 15 minutes; in this period about 30 pseudopodia are extended. Previously we have presented the cell trajectories for several strains and developmental stages [12] (see also Fig. S1). The mean square displacement as a function time, 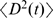, exhibits a slow approach to a linear function (Fig. 3A), which is typical for a transition of a correlated random walk at short times to a Brownian random walk after longer times [6], [23]. Previously, the often used equation for a correlated random walk were fit to the data points to estimate persistence time and speed of the cells [12]. The aim of the present study is to analyze the mechanism of cell movement from the perspective of the extending pseudopodia, which have a specific length and direction. A correlated random walk in two dimensions can also be described with steps and turns [24], [25]. With the replacement of the number of steps (n) in Eq. 7 in reference [25] for n = Ft we obtain
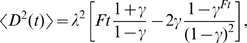
(4)where *λ* is the step size in µm, F is the step frequency, and *γ* is the correlation factor of dispersion (0<*γ*<1), defined as the arithmetic mean of the cosine of the turn angle *θ* between steps
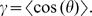
(5)With three variables (F, *λ*, *γ*) the estimates of the parameters become uncertain. Fortunately, the step size can be deduced accurately from experimental data. As will be shown below in Eq. 10, the step size is given by *λ* = *λ_p_*cos(*ϕ*/2), where measurements for *λ_p_* and *ϕ* are presented in Table 1. Using this value for *λ*, the dispersion data were fitted to obtain the observed correlation factor of dispersion (*γ*_obs_) with the corresponding turn angle (*θ*). In cells starved for 1 or 3 hours the correlation factor is only ∼0.5 with turn angle of ∼60 degrees. At 5 and 7 hours of starvation, cells move with much stronger persistence (correlation factor of 0.74 and 0.81 and a small turn angle of 42 and 36 degrees). Deletion of PLA2 or guanylyl cyclases prevents this increase of correlation factor, persistence is very low and cells disperse poorly.

How is pseudopod extension related to the observed correlation factor of dispersion *γ*_obs_? As previously stated (see Fig. 2), *Dictyostelium* cells may extend either *de novo* pseudopodia in nearly random directions, or splitting pseudopodia in a direction similar to the previous direction. Therefore, cells that extend exclusively de novo pseudopodia are expected to exhibit a random walk with *γ*_obs_ = 0 (turn angle *θ* = 90 degrees), whereas cells extending exclusively splitting pseudopodia will exhibit strong persistence with large *γ* and small turn angle *θ*. As a consequence, *γ*_obs_ is expected to depend on the ratio *s* of splitting/de novo pseudopodia. Fig. 3B demonstrates that within experimental error this relationship is approximately linear; this holds true for the mutants as well as for wild type cells at different stages of development. The linear regression of all data yields *γ*_obs_ = 0.921*s*−0.044. Thus, when all pseudopodia are de novo (*s* = 0) the correlation factor is small (*γ*_obs_ = −0.044) giving a turn angle 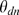 = 93 degrees, close to the expected value of 90 degrees for random turns. In contrast, when all pseudopodia are the result of splitting (*s* = 1) the correlation factor is large (*γ*_obs_ = 0.88) yielding a small turn angle (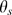 = 29 degrees). The implication of small turn angles for splitting pseudopodia will be discussed later.

### Model for persistent movement

The alternating right/left extension of splitting pseudopodia can be used to simplify a description of the movement of *Dictyostelium* cells over longer distances. In this approach, the simplification may be valid for movement on a longer time scale only, as we study here, but may not be appropriate over shorter time scales of a few pseudopodia. Because pseudopodia are frequently extended alternating right/left, we consider movement by pairs of two pseudopodia.

**Figure 4:**
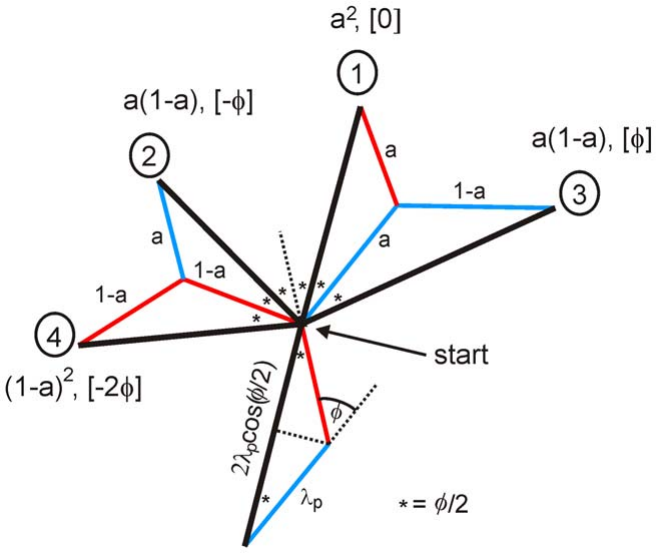
Bipedal amoeboid movement. The diagram shows the probabilities, angles and sizes of pairs of pseudopod extensions. *Dictyostelium* cells frequently extend alternating right/left splitting pseudopodia. Pseudopodia to the left are in red, to the right in blue, and the movement after two pseudopodia is in black. Indicated are the four possible movements with two splitting pseudopodia after the cell has made a right and left pseudopod. The probability for alternating right/left or left/right is (*a*), while the probability for consecutive right/right or left/left is (1-*a*), yielding the probabilities of the four pseudopod pairs. The angle between pseudopodia is *ϕ*.

Figure 4 shows four possibilities of pairs of splitting pseudopodia, which are the RL, LR, RR and LL, each with corresponding probabilities and angles as indicated. In addition to these splitting pairs, three combinations with de novo pseudopodia are possible: split-de novo, de novo-split, and de novo-de novo. The correlation factor of dispersion yields for the seven pairs:
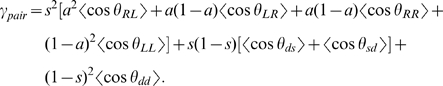
(6)De novo pseudopodia are extended in a random direction, i.e. 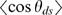, 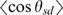 and 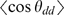 equal zero. The turn angles of the four splitting pairs are 0, *ϕ* and 2*ϕ*, as indicated in Fig. 4A, and the variance is approximately 2*σ_ϕ_* ^2^ (see Fig. 2B). Consequently Eq. 6 reduces to:
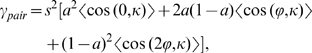
(7)where 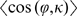 denotes the expected value of the cosines of the angles on a circle with weights given by the vMD with mean *ϕ* and variance given by *κ* = 1/(2*σ_ϕ_* ^2^). Since all splitting pseudopodia show the same variance this can be further reduced to
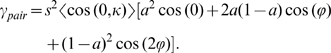
(8)In this equation 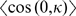 is obtained by calculating the probabilities of all turn angles on a circle with the vMD using Eqs. 1 and 2 and then taking the weighted average of the cosines of these angles. Although this procedure is straightforward, Eq. 8 can be further simplified, because for *σ_ϕ_* smaller than ∼50 degrees a good approximation is 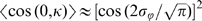 (see Fig. S2). Finally, on a longer time scale and averaged over many steps, the correlation factor of pairs is related to the correlation factor of its underlying two steps by 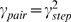. With these replacements we obtain the analytical expression for the correlation factor
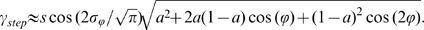
(9)Thus, the correlation factor *γ* is the product of three terms: the splitting ratio *s*, a noise term with the variance *σ_ϕ_*, and a term with right/left bias *a* and angle *ϕ*.

Finally, by considering movement in pairs of steps, Fig. 4 reveals that the step size of the displacement is given by
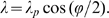
(10)


### Monte Carlo simulations of cell movement

We used Monte Carlo simulations to investigate how *λ* and *γ* depend on the pseudopod parameters size *λ_p_*, splitting fraction *s*, alternating ratio *a*, angle between split pseudopodia *ϕ* and variance of this angle *σ_ϕ_* ^2^. These simulations are also useful to inspect whether step size *λ* and correlation factor *γ* are correctly described by Eqs. 8–10. The direction in which a pseudopod is extended appears to be an ordered stochastic event [12] that depends on multiple decisions according to the following scheme: The next pseudopod is a splitting or de novo according to the ratio *s*. A splitting pseudopod is extended with angle *ϕ* to the right or left relative to the previous splitting according to the alternating right/left bias *a*. A de novo pseudopod is extended in a random direction. The splitting pseudopod that appears after a de novo pseudopod has an equal probability to be extended to the left or right. Finally, the direction of the emerging pseudopod has a variance *σ_ϕ_* ^2^. The Monte Carlo simulation starts with a random angle *α(1)* of the first pseudopod and then uses the probabilities for splitting fraction *s*, alternating ratio *a*, angle between split pseudopodia *ϕ* and variance of this angle *σ_ϕ_* ^2^ to stochastically simulate the angle of the next pseudopod (see methods and Table 1 for pseudopod parameters of wild type and mutant cells). The simulated trajectories are qualitatively similar to the experimentally observed trajectories (see Fig. S1B): Fed wild type cells or mutants with abundant de novo pseudopodia make many turns and have small displacement, whilst the trajectories of starved wild type cells with abundant pseudopod splitting are more persistent with large displacements.

**Figure 5:**
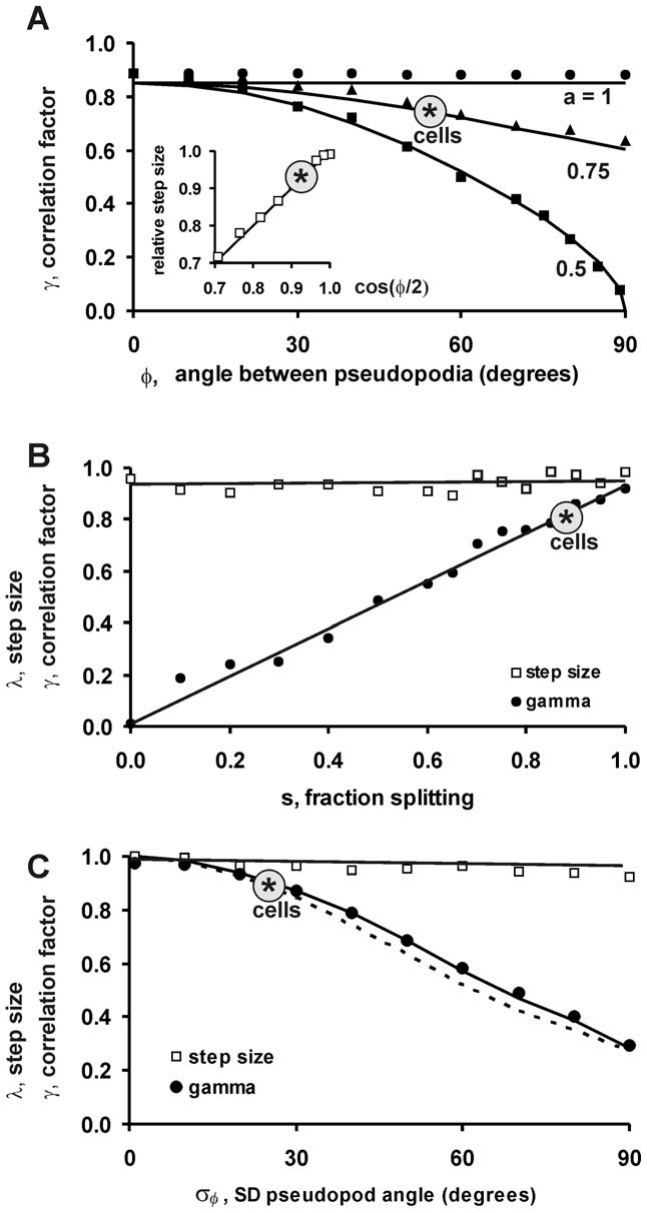
Predicted correlation factor of persistence and step size. The trajectories of 100,000 cells were obtained by Monte Carlo simulation; the displacement was analyzed with Eq. 4 to obtain the correlation *γ*_MC_ and step size *λ* (in units of pseudopod size and thus dimensionless). Simulations were performed with the parameter values as indicated on the x-axis, while the other parameters had the following standard values *λ*_p_ = 1; *s* = 1; *a* = 1; *ϕ* = 55 degrees; *σ_ϕ_* = 28 degrees. The data points denote the outcome of the Monte Carlo simulations, the lines are generated using Eq. 9, while the dotted line in panel C is generated using Eq. 8. The asterisks represent the predicted value for 5h starved *Dictyostelium* cells. A. The effect of angle *ϕ* and right/left bias *a* (*a* = 1, all pseudopodia alternating right/left; *a* = 0.5 right/left is random). B. The effect of the fraction of splitting pseudopodia (*s*). C. The effect of the variance of the angle between pseudopodia (*σ_ϕ_*).

To investigate how the correlation factor *γ* depends on pseudopod parameters, the displacement 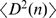 was calculated from 100,000 trajectories obtained by MC simulation using a unit pseudopod size and different values of *s*, *a*, *ϕ* and *σ_ϕ_*. The obtained displacement 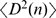 was then fitted to Eq. 4 to obtain estimates for the step size *λ* and Monte Carlo correlation factor, *γ*_MC_. The symbols in Fig. 5 show the results of the MC simulation, whereas the curves are the result of Eqs. 8–10.

We first investigated the angle *ϕ* between splitting pseudopodia and the alternating right/left bias *a*. When all splitting pseudopodia are alternating (*a* = 1), the cells make a nearly perfect zig-zag trajectory, and therefore the angle *ϕ* has very little effect on the persistence factor *γ* (Fig. 5A). When splitting pseudopodia are extended in a random fashion to the right or left (*a* = 0.5), the persistence factor *γ*_MC_ decreases sharply as *ϕ* becomes larger than ∼30 degrees. At an intermediate right/left bias (*a* = 0.75) the persistence factor *γ*_MC_ remains relatively high as long as the angle between pseudopodia is below 60 degrees. The results of the MC simulation appear to be described very well by the simplified model (Eqs. 8–10). Furthermore, at the observed angle of *ϕ* = 55 degrees and alternating factor of *a* = 0.77, the deduced persistence factor *γ*_MC_ is 0.88 (see asterisk in Fig. 5A).

The fraction of splitting pseudopodia has a major impact on the persistence factor *γ*. In the MC simulations, the value of *γ* declines approximately linearly with the value of *s* (Fig. 5B), as was also observed experimentally (Fig. 3B), and obtained in Eqs. 8 and 9. Finally, we investigated the contribution of the variance *σ_ϕ_* ^2^ of the splitting angle to the persistence factor *γ*. This reveals that the persistence decreases strongly with increasing variance (Fig. 5C), with *γ*_MC_ following an approximately linear relationship with cos(*σ_ϕ_*). The MC simulations are well described by Eq. 8, but deviate from Eq. 9 at *σ_ϕ_*>30 degrees, as expected (see Fig. S2).

We also used these Monte Carlo simulations to obtain an estimate of the step size *λ*. It appears that *λ* does not to depend on *s* and *a*, but depends on *ϕ* according to 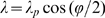 (Inset Fig 5A), as was obtained in Eq. 10.

In summary, the obtained correlation factor from the MC simulation (*γ*_MC_) are nearly identical to the correlation factor calculated with Eq. 9 (*γ*_step_). This suggests that the movement of *Dictyostelium* cells is qualitatively and quantitatively described very well by the model of persistent steps and random turns with the observed pseudopod parameters *λ_p_*, *s*, *a*, *ϕ* and *σ_ϕ_*.

### Movement of the centroid of the cell

How does the movement of pseudopodia relate to the movement of the centroid of the cell? The data presented in Table 1 reveal that the observed correlation factor *γ*_obs_ of the centroid for different cell types correspond well with the deduced correlation factors of the pseudopods (*γ*_MC_ and *γ*_step_), but is always larger by ∼15% (Table 1). Apparently, the observed turn angle of the cell's centroid is smaller than the turn angle of the extending pseudopod. Inspection of movies of 5h starved AX3 cells confirm this notion: the average angle between splitting pseudopodia is 55±28 degrees (Fig. 2A), while the centroid moves during period at an angle of only 31±23 degrees (mean and SD). Equation 9 reveals that the correlation factor *γ*_step_ increases by 15% when *ϕ* = 55±28 degrees for the pseudopod is replaced by *ϕ* = 31±23 degrees for the cells centroid.

Probably two phenomena are responsible for the difference between pseudopod and centroid: extension of multiple pseudopodia and geometry of cells. When cells extend multiple pseudopodia it is likely that at any given instant of time, the front of the cell moves with a fixed fraction of the vector sum of velocities possessed by the pseudopodia active at that instant in time. The temporal overlap of two pseudopodia was deduced from the measured probability distributions of pseudopod extensions (Fig 2F in [26]), which reveal that ∼25% of the pseudopodia overlap with another pseudopod during on average ∼40% of their extension time. This suggest that the tip of the cell moves at an angle that is ∼6 degrees smaller than 55 degrees. Secondly, geometry predicts that the rear of the cell makes smaller changes of direction than the tip of the cell, comparable to the differences in curvature made by the front and rear wheels of a car. Figure S3 indicates that for a stereotypic pseudopod at 55 degrees the directional change of the centroid is ∼40 degrees (see Fig. S3). Together, multiple pseudopodia and cell geometry can explain observed difference between pseudopod and centroid changes of direction, leading to the small 15% difference between deduced pseudopod correlation factor (*γ*_MC_ and *γ*_step_) and observed centroid correlation factor (*γ*_obs_).

### Directional displacement

**Figure 6:**
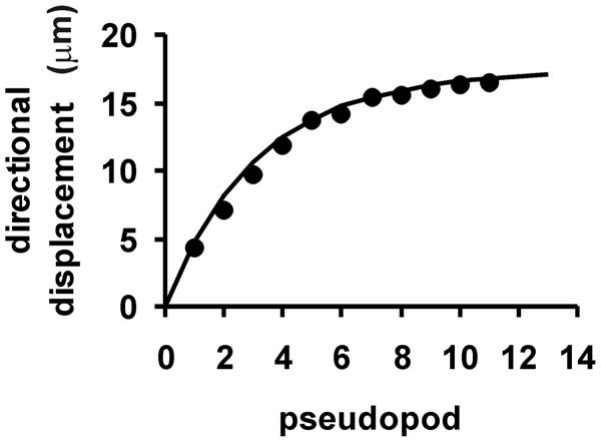
Directional displacement. Pseudopod formation and trajectories were recorded for 5h starved *Dictyostelium* cells; 8 cells were followed during 15 min. The directional displacement is the distance moved after n pseudopodia in the direction of the first pseudopod. Data points are the means of ∼120 measurements, the line is the outcome of Eq. 11 with pseudopod parameters as indicated in Table 1.

The *directional* displacement 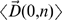 is the displacement after n steps in the direction of the first step. An expression for the directional displacement is especially relevant when the organism is exposed to positional cues leading to a drift in one direction, such as during chemotaxis. The directional displacement of a cell after extending one pseudopod at an angle *θ* is 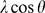, and for a population of cells 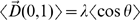. By Eqs. 3 and 10, the displacement at the first step may be written as 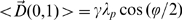, and at the i^th^ step 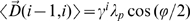, see Eq. 6 of reference [25]. The cumulative displacement after n steps is
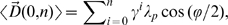
(11a)which at 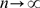 is given by
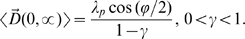
(11b)In essence, this equation describes the displacement of a cell population in which all cells extend the first pseudopod in the same direction. Subsequent pseudopodia are extended with a bias, which reduces geometrically with each step; the correlation factor *γ* indicates how many pseudopodia have correlated direction and therefore how far the population will disperse in the direction of the first pseudopod. Figure 6 presents the directional displacement as observed experimentally in wild type cells. The displacement in the direction of the first pseudopod slowly decreases at each subsequent pseudopod, approaching random movement after ∼10 pseudopodia. On average a cell moves ∼15 µm in the direction of the first pseudopod, which is the equivalent of about 3 pseudopodia (given a pseudopod size of ∼5 µm). This figure also presents the directional displacement as modeled by Eq. 11a with observed data for *λ*_p_, *ϕ* and *γ*, which is in very close agreement with experimental data, again suggesting that the movement of a cell is satisfactory described by the model with five pseudopod parameters.

### Cell shape, the variance in the direction of pseudopod extension, and dispersal

The variation in pseudopod direction *σ_ϕ_* ^2^ plays an important role in Eqs. 8–11 describing cell dispersal. Previously [12] we have shown that the next pseudopod emerges at a specific distance *d* from the tip of the current pseudopod, and is then extended perpendicular to the cell surface (i.e. perpendicular to the tangent to the surface curvature at the position where the pseudopod emerges). The pseudopod direction is expected to have high confidence for cells with a smooth ellipsoid shape, because the local bending is very predictable. However, this confidence is much smaller for cells with a very irregular shape. We investigated the role of cell shape using three experiments. First we demonstrate that the variance *σ_ϕ_* ^2^ indeed depends on the variance of the tangent and the normal to the tangent. Second, we show that wild type or mutant cells with irregular shape exhibit increased variance *σ_ϕ_* ^2^. Finally we show that, due to the increased variance, the mutant exhibits poor dispersal.

Quimp3 was used to construct the tangent to the surface curvature at the position where the pseudopod emerges. We first determined for wild-type cells the angle *α_t_* of this tangent relative to the previous pseudopod (*α_t_* = 34.5±24.9 degrees), and the angle *β* of the new pseudopod relative to this tangent (*β* = 89.1±13.3 degrees). As mentioned above, the observed angle of the new pseudopod relative to the previous pseudopod is *ϕ* = 55.2±27.8 degrees. We expect that the angle of the tangent relative to the previous pseudopod is independent from the angle of the pseudopod relative to the tangent; therefore we expect 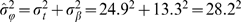. Indeed, the observed standard deviation of 27.8 degrees is close to this expected value of 28.2 degrees. Importantly, the largest contribution to *σ_ϕ_* ^2^ is derived from the variance of the tangent *σ_t_* ^2^, which is related to the local shape of the cell.

**Figure 7:**
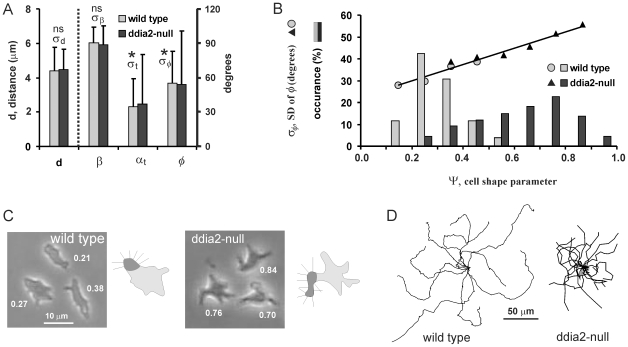
Movement of a mutant with irregular shape. Pseudopod formation and trajectories were recorded for 5h starved wild type and mutant *ddia2*-null cells. For the next pseudopod we measured the angle *ϕ* and distance *d* relative to the current pseudopod, the angle *α*_t_ of the tangent relative to the current pseudopod and the angle *β* of the pseudopod relative to the tangent. **A**. The data show the means and SD measured for 312 and 289 pseudopodia from wild type and *ddia2*-null cells, respectively. The means are not significantly different between wild type and *ddia2*-null cells, but the SD *σ_ϕ_* and *σ*_t_ are significantly larger for *ddia2*-null compared to wild type (Chi-square, P<0.0001); the SD *σ_d_* and *σ_β_* are not significantly different. **B**. The shape parameter *Ψ* was determined at the moment of pseudopod extension. Bars show the frequency distributions for 312 wild type and 289 *ddia2*-null cells. Data points show *σ_ϕ_*, the SD of *ϕ*. Linear regression analysis of all data point yields *σ_ϕ_*. = 37.6*Ψ*+22.8; R^2^ = 0.965. **C**. Pictures showing three representative cells; numbers indicate the measured values of *Ψ*. Mutant *ddia2*-null has an irregular star-like shape, while wild-type cells are smoother and more spherical. Each diagram shows a cell with a pseudopod (dark grey area); the small lines indicate the direction of pseudopodia that emerge by pseudopod splitting perpendicular to the surface. Due to the irregular shape, pseudopodia extending by *ddia2*-null cells exhibit more angular variation. **D**. Displacement of wild type and *ddia2*-null cells during 15 minutes.

In the collection of *Dictyostelium* mutants, we selected a strain with an irregular shape. Mutant *ddia2*-null with a deletion of the *forH* gene encoding the formin dDia2 has a star-like shape (Fig. 7C). In this mutant, new pseudopodia are extended at about the same frequency and distance from the present pseudopodia as in wild type cells, pseudopodia also grow perpendicular to the surface, and are extended roughly in the same direction of *ϕ* = 55 degrees as wild type cells (Fig. 7A). However pseudopodia exhibit much more variation in direction (*σ_ϕ_* = 47 degrees compared to *σ_ϕ_* = 28 degrees for wild type cells). Finally, we determined a shape parameter *Ψ* that indicates how much the cell outline deviates from an ellipse (see method section and Fig. S4). Figure 7B reveals that cells with increased irregular shape, either being wild-type or mutant, exhibit strongly increased variance *σ_ϕ_* ^2^. Importantly, the distance *d* and angle *ϕ* of the pseudopodia does not change with cell shape (Fig. 7A).

Using the observed values for *s*, *a*, *ϕ*, and *σ_ϕ_* for *ddia2*-null cells we expect from Eq. 9 to obtain *γ*_step_ = 0.43, significantly lower compared to *γ*_step_ = 0.69. for wild type cells. Fig. 7D shows that the dispersion of *ddia2*-null cells is strongly reduced. The observed mean square dispersion was fitted to Eq. 4 yielding a correlation factor of *γ*_obs_ = 0.53 (Table 1), close to the value that was predicted from the extension of pseudopodia from an irregular surface.

In summary, these and previous results [12] suggest that a splitting pseudopod is induced at some distance *d* from the tip of the current pseudopod, and then grows perpendicular to the surface. In a cell with a regular shape, the tangent and therefore pseudopod direction can be approximated using the distance *d*; alternating R/L extensions lead to a relative straight zig-zag trajectory, providing strong persistence of movement. In a cell with a very irregular shape, the local curvature of the membrane at distance *d* is unpredictable. Consequently, alternating R/L splitting occur with large variation of directions, leading to frequent turns and poor persistence.

## Discussion

The movement of many organisms in the absence of external cues is not purely random, but shows properties of a correlated random walk. The direction of future movement is correlated with the direction of prior movement. For organisms moving in two dimensions, such as most land-living organisms, this implies that movement to the right is balanced on a short term by movement to the left to assure a long-term persistence of the direction. In bipedal locomotion, the alternating steps with the left and right foot will yield a persistent trajectory. Amoeboid cells in the absence of external cues show ordered extension of pseudopodia: a new pseudopod emerges preferentially just after the previous pseudopod has stopped growth [12]. Importantly, the position at the cell surface where this new pseudopod emerges is highly biased. When the current pseudopod has been extended to the left (relative to the previous pseudopod), the next pseudopod emerges preferentially nearby the tip at the right side of the current pseudopod. Since pseudopodia are extended perpendicular to the cell surface, this next pseudopod is extended at a small angle relative to the current pseudopod [12]. Therefore, this (imperfect) alternating right/left pseudopod splitting resembles bipedal locomotion. Cells may also extend a de novo pseudopod somewhere at the cell body, which is extended in a random direction. In starved *Dictyostelium* cells, the probability of extending a de novo pseudopod is ∼10-fold lower than of pseudopod splitting (probability calculated per µm circumference of the cell [12]).

The model for pseudopod-based cell dispersion depends on five parameters, the pseudopod size (*λ*), the fraction of split pseudopodia (*s*), the alternating left/right bias (*a*), the angle between pseudopodia (*ϕ*) and the variance of this angle (*σ_ϕ_* ^2^). With these parameters the experimental data on mean square displacement and directional displacement are well-explained using Eqs. 9 and 11, respectively. Pseudopodia are the fundamental instruments for amoeboid movement. The notion that the trajectories are described well by the five pseudopod parameters probably implies that we have identified the basic concept of the amoeboid correlated random walk: persistent alternating pseudopod splitting and formation of de novo pseudopodia in random directions.

The cells may modify one or more of these five pseudopod parameters in order to modulate the trajectories (see Table 1). Nearly all mutants, as well as wild type cells at different stages of starvation and development, have approximately the same average pseudopod size *λ_p_*. In addition, the alternating right/left bias (*a*) fluctuates between 0.67 and 0.82, and the angle between splitting pseudopodia (*ϕ*) between 50 and 62 degrees. [*pla2*-null cells are the only exception [12]; emerging pseudopodia in *pla2*-null cells exhibit longer growth periods (∼27 s) than wild type cells (∼13 s), and are thus longer]. This suggests that all strains use the same mechanism for pseudopod splitting. In contrast to these constant *properties* of split pseudopodia, the *fraction* of split pseudopodia (*s*) changes dramatically upon starvation, and appears to be regulated by cGMP and PLA2 signaling. Well-fed cells extend pseudopodia that are predominantly de novo in random directions, leading to a nearly Brownian random walk [20]. Upon starvation, the appearance of cGMP and PLA2 signaling enhances splitting and suppresses de novo pseudopod extensions, which leads to more persistent movement. The important role of the fraction of splitting pseudopodia for cell movement is also depicted by the linear dependence of the correlation factor *γ* on the fraction *s* of splitting pseudopodia (Fig. 3B and Eq. 9).

The variance of the angle of pseudopod extension (*σ_ϕ_* ^2^) plays an important role in movement. In wild type cells, as well as in many mutant strains, *σ_ϕ_* is about 28 degrees. The primary source of the variance of pseudopod angles lies in the variation of cell shape, by which the normal to the cell surface at a specific position on this surface will have significant variation. Since the direction of pseudopodia is given by this normal, it is predicted that a cell with irregular shape should have more variation in pseudopod direction, and consequently shows poor dispersion. The experiments with mutant *ddia2*-null cells strongly support this interpretation. Wild-type cells have a relatively regular spherical shape by which two nearby pseudopodia are extended in nearly the same direction (small *σ_ϕ_*). In contrast, mutant *ddia2*-null cells have an irregular star-like shape; therefore, two nearby pseudopodia are often extended in very different directions (large *σ_ϕ_*). The variance *σ_ϕ_* ^2^ can be regarded as the noise of the system. It indicates how fast a cell that extends only alternating splitting pseudopodia (*a* = 1 and *s* = 1) will lose correlation of directionality. With *σ_ϕ_* = 28 degrees for wild type cells it follows from Eq. 10 that after ten pseudopodia the correlation of direction is still ∼0.5. In contrast, for *ddia2*-null cells we obtained *σ_ϕ_* = 46.5 degrees, which implies that already after four pseudopodia the correlation of direction has declined to ∼0.5. Supported by Monte Carlo simulations using the parameters of the mutant, we conclude that poor dispersion of *ddia2*-null cells is due to the increased variance of pseudopod angles, which is caused by its irregular shape.

The correlation factor *γ* is the product of three terms (see Fig. 5 and Eq. 9), namely: splitting fraction (*s*), alternating pseudopod angles (*a* and *ϕ*), and the SD of the pseudopod angle (*σ_ϕ_*). Strong persistence of cell movement is attained when all three terms are large and about equal in magnitude. Starved wild type cells follow this strategy: each term is ∼0.9, resulting in the observed correlation factor of 0.74. Mutants in which one of these terms is compromised, such as reduced splitting in *sgc/pla2*-null cells or enhanced noise of *ddia2*-null cells, have poor dispersion.

In summary, the correlated random walk of amoeboid cells is well described by the balanced bipedal movement, mediated by the alternating right/left extension of splitting pseudopodia. Cells deviate from movement in a straight line due to noise and because cells occasionally hop or make random turns. The turns in particular are used by the cells to modulate the persistence time, thereby shifting between nearly Brownian motion during growth and strong persistent movement during starvation.

## Supporting Information

Figure S1 Trajectories. Movies were recorded during 15 minutes and the trajectories of the centroid of ten cells were determined. **A**. Wild type Dictyostelium cells at different times after removing of food. The frequency and size of pseudopod extension is not very different, but starved cells extend predominantly splitting pseudopodia (see (16)). **B**. Trajectories of 5 hour starved mutant cells with deletions of genes encoding guanylyl cyclases (sGC and GCA) and PLA2. **C**. Monte Carlo simulations of the trajectories calculated with the pseudopod parameters that were obtained experimentally for the mutants as indicated in Table 1.
(0.07 MB PDF)

Figure S2Analysis of the noise equation 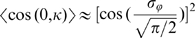. Pseudopodia are extended with a variance σ*_ϕ_^2^*. In this equation, the notation 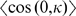 is the average of the cosines of the angles on a circle with weights given by the von Mises Probability Distribution (vMD) with mean of 0 degrees and variance given by *κ* = 1/*σ_ϕ_* ^2^. The figure reveals that 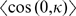 deviates less than 2% from the simple expression 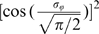 for values of *σ_ϕ_*<40 degrees.(0.03 MB PDF)

Figure S3 Movement of pseudopod and centroid of a cell. A. The cell is drawn as an ellipse with short and long axes of 3 and 6 µm, respectively. A pseudopod of 5 µm is extended perpendicular to the ellipse at 55 degrees relative to the long axes of the ellipse, which define the starting point and direction of the pseudopod. The position of the centroid is indicated by an asterisk. **B**. In *Dictyostelium* cells a pseudopod usually extends during ∼12 seconds, and then the cytoplasm moves into the pseudopod and the rear is retracted. The open headed arrows indicate that the front of the cell moves to the tip of the pseudopod, and the rear of the cell moves in the direction of the old axis of the cell. **C**. Schematic after a few Right/Left pseudopod extensions. The centroid makes smaller turns than the pseudopod, ∼40 degrees.
(0.07 MB PDF)

Figure S4 Determination of the shape parameter *Ψ*. The cell outline is used to draw two ellipses, the largest possible ellipse inside the cell and the smallest possible ellipse outside the cell. Then an intermediate ellipse is constructed by interpolation of the inner and outer ellipse; the figure shows the intermediate ellipse. This ellipse intersects the outline, thereby forming the blue areas *O* of the cell that are outside the intermediate ellipse, and yellow areas *I* of the intermediate ellipsoid that do not belong to the cell. The intermediate ellipse was interpolated in such a way that *O* = *I*. The parameter of cell shape is defined as *Ψ* = (*O*+*I*)/*T*, where *T* is the surface area of the cell (grey + blue). The minimal value is *Ψ* = 0 when the cell is an ellipse, and the maximal value is *Ψ* = 2 for a cell with extreme long extensions; the maximal value observed among ∼600 *Dictyostelium* cells is *Ψ* = 0.92. The neuron cell is shown for comparison.
(0.13 MB PDF)
