# Supplementary material for: A Model for a Correlated Random Walk Based on the Ordered Extension of Pseudopodia
Source: PLoS Comput Biol. 2010 Aug 12;6(8):e1000874. doi: 10.1371/journal.pcbi.1000874 (PMC2920832; doi:10.1371/journal.pcbi.1000874)
Supplement: Figure S1 — Trajectories. Movies were recorded during 15 minutes and the trajectories of the centroid of ten cells were determined. A. Wild type Dictyostelium cells at different times after removing of food. The frequency and size of pseudopod extension is not very different, but starved cells extend predominantly splitting pseudopodia (see (16)). B. Trajectories of 5 hour starved mutant cells with deletions of genes encoding guanylyl cyclases (sGC and GCA) and PLA2. C. Monte Carlo simulations of the trajectories calculated with the pseudopod parameters that were obtained experimentally for the mutants as indicated in Table 1. (0.07 MB PDF) [file pcbi.1000874.s001.pdf]

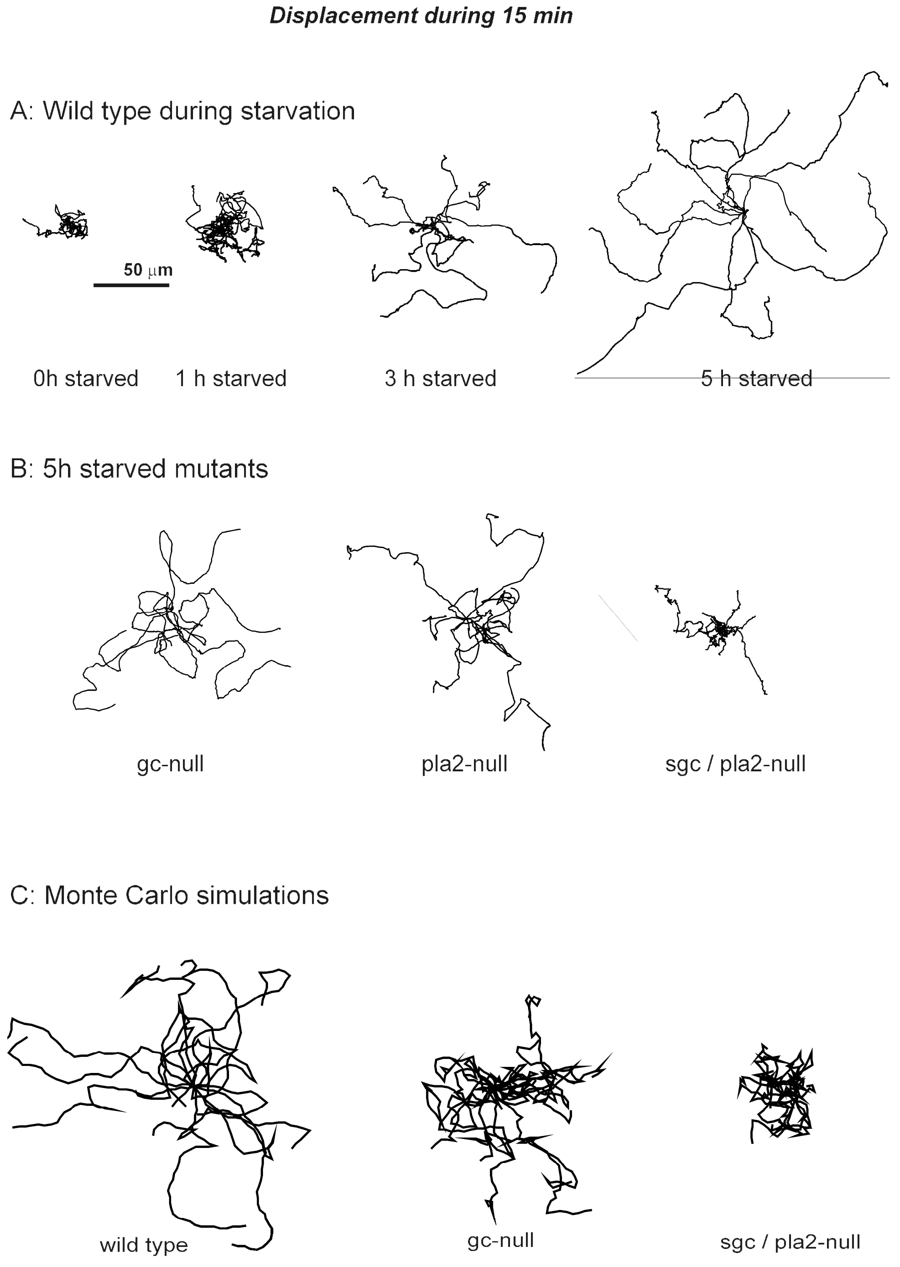

Figure S1. Trajectories. Movies were recorded during 15 minutes and the trajectories of the centroid of ten cells were determined. **A.** Wild type Dictyostelium cells at different times after removing of food. The frequency and size of pseudopod extension is not very different, but starved cells extend predominantly splitting pseudopodia (see (16)). **B.** Trajectories of 5 hour starved mutant cells with deletions of genes encoding guanylyl cyclases (sGC and GCA) and PLA2. **C.** Monte Carlo simulations of the trajectories calculated with the pseudopod parameters that were obtained experimentally for the mutants as indicated in Table I.
