# Supplementary material for: A Model for a Correlated Random Walk Based on the Ordered Extension of Pseudopodia
Source: PLoS Comput Biol. 2010 Aug 12;6(8):e1000874. doi: 10.1371/journal.pcbi.1000874 (PMC2920832; doi:10.1371/journal.pcbi.1000874)
Supplement: Figure S2 — Analysis of the noise equation . Pseudopodia are extended with a variance σϕ2. In this equation, the notation is the average of the cosines of the angles on a circle with weights given by the von Mises Probability Distribution (vMD) with mean of 0 degrees and variance given by κ = 1/σϕ 2. The figure reveals that deviates less than 2% from the simple expression for values of σϕ<40 degrees. (0.03 MB PDF) [file pcbi.1000874.s002.pdf]

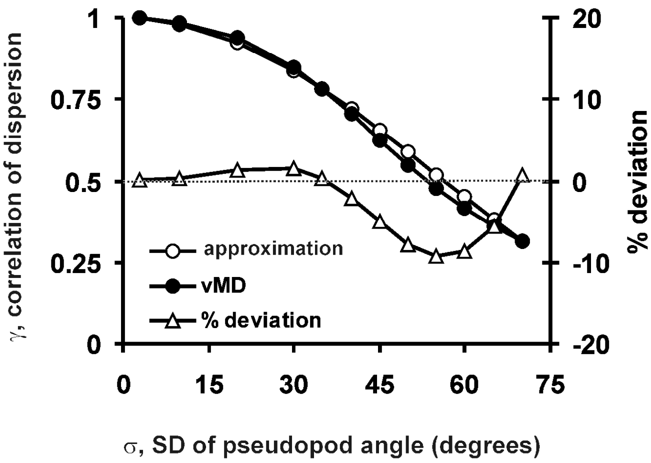

Figure S2. Analysis of the noise equation $\langle\cos(0,\kappa)\rangle \approx [\cos(\frac{\sigma_\phi}{\sqrt{\pi/2}})]^2$

Pseudopodia are extended with a variance σ_ϕ^2. In this equation, the notation $\langle\cos(0,\kappa)\rangle$ is the average of the cosines of the angles on a circle with weights given by the von Mises Probability Distribution (vMD) with mean of 0 degrees and variance given by $\kappa = 1/\sigma_\phi^2$. The figure reveals that $\langle\cos(0,\kappa)\rangle$ deviates less than 2% from the simple expression $[\cos(\frac{\sigma_\phi}{\sqrt{\pi/2}})]^2$ for values of $\sigma_\phi < 40$ degrees.
