# Supplementary material for: A Model for a Correlated Random Walk Based on the Ordered Extension of Pseudopodia
Source: PLoS Comput Biol. 2010 Aug 12;6(8):e1000874. doi: 10.1371/journal.pcbi.1000874 (PMC2920832; doi:10.1371/journal.pcbi.1000874)
Supplement: Figure S3 — Movement of pseudopod and centroid of a cell. A. The cell is drawn as an ellipse with short and long axes of 3 and 6 µm, respectively. A pseudopod of 5 µm is extended perpendicular to the ellipse at 55 degrees relative to the long axes of the ellipse, which define the starting point and direction of the pseudopod. The position of the centroid is indicated by an asterisk. B. In Dictyostelium cells a pseudopod usually extends during ∼12 seconds, and then the cytoplasm moves into the pseudopod and the rear is retracted. The open headed arrows indicate that the front of the cell moves to the tip of the pseudopod, and the rear of the cell moves in the direction of the old axis of the cell. C. Schematic after a few Right/Left pseudopod extensions. The centroid makes smaller turns than the pseudopod, ∼40 degrees. (0.07 MB PDF) [file pcbi.1000874.s003.pdf]

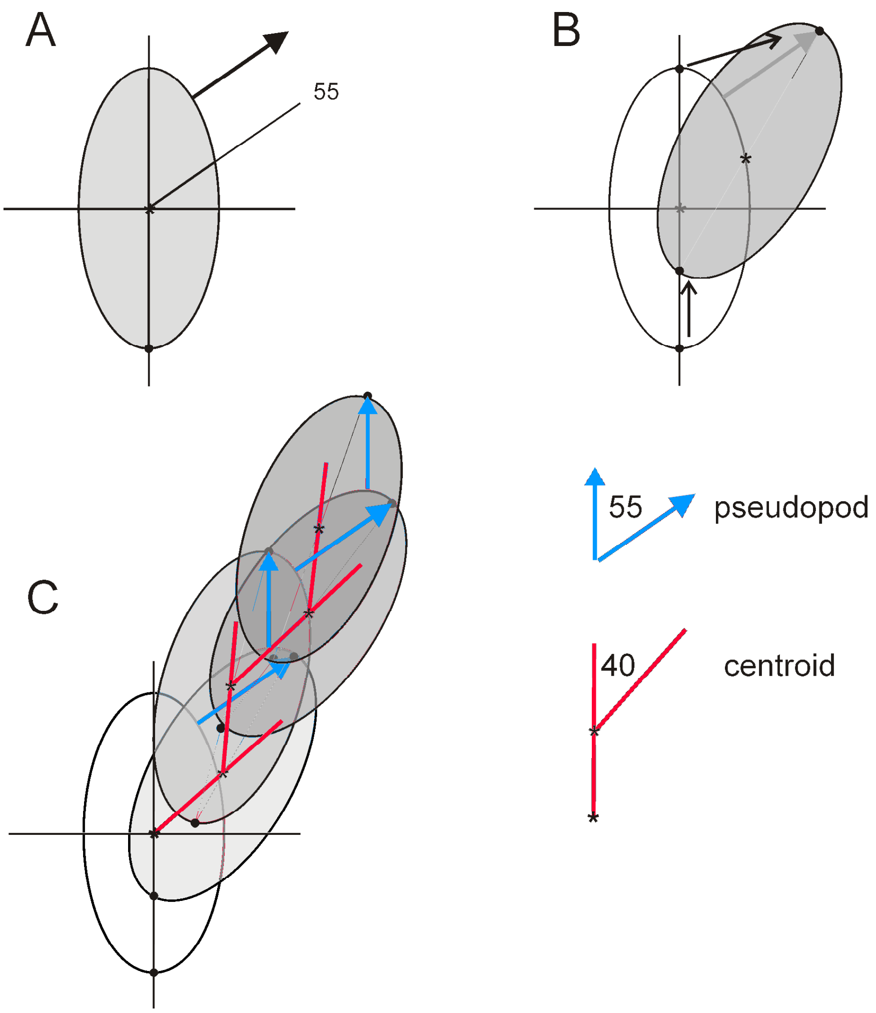

Figure S3. Movement of pseudopod and centroid of a cell. A. The cell is drawn as an ellipse with short and long axes of 3 and 6 µm, respectively. A pseudopod of 5 µm is extended perpendicular to the ellipse at 55 degrees relative to the long axes of the ellipse, which define the starting point and direction of the pseudopod. The position of the centroid is indicated by an asterisk. **B**. In *Dictyostelium* cells a pseudopod usually extends during ~12 seconds, and then the cytoplasm moves into the pseudopod and the rear is retracted. The open headed arrows indicate that the front of the cell moves to the tip of the pseudopod, and the rear of the cell moves in the direction of the old axis of the cell. **C**. Schematic after a few Right/Left pseudopod extensions. The centroid makes smaller turns than the pseudopod, ~40 degrees.
