# Supplementary material for: A Model for a Correlated Random Walk Based on the Ordered Extension of Pseudopodia
Source: PLoS Comput Biol. 2010 Aug 12;6(8):e1000874. doi: 10.1371/journal.pcbi.1000874 (PMC2920832; doi:10.1371/journal.pcbi.1000874)
Supplement: Figure S4 — Determination of the shape parameter Ψ. The cell outline is used to draw two ellipses, the largest possible ellipse inside the cell and the smallest possible ellipse outside the cell. Then an intermediate ellipse is constructed by interpolation of the inner and outer ellipse; the figure shows the intermediate ellipse. This ellipse intersects the outline, thereby forming the blue areas O of the cell that are outside the intermediate ellipse, and yellow areas I of the intermediate ellipsoid that do not belong to the cell. The intermediate ellipse was interpolated in such a way that O = I. The parameter of cell shape is defined as Ψ = (O+I)/T, where T is the surface area of the cell (grey + blue). The minimal value is Ψ = 0 when the cell is an ellipse, and the maximal value is Ψ = 2 for a cell with extreme long extensions; the maximal value observed among ∼600 Dictyostelium cells is Ψ = 0.92. The neuron cell is shown for comparison. (0.13 MB PDF) [file pcbi.1000874.s004.pdf]

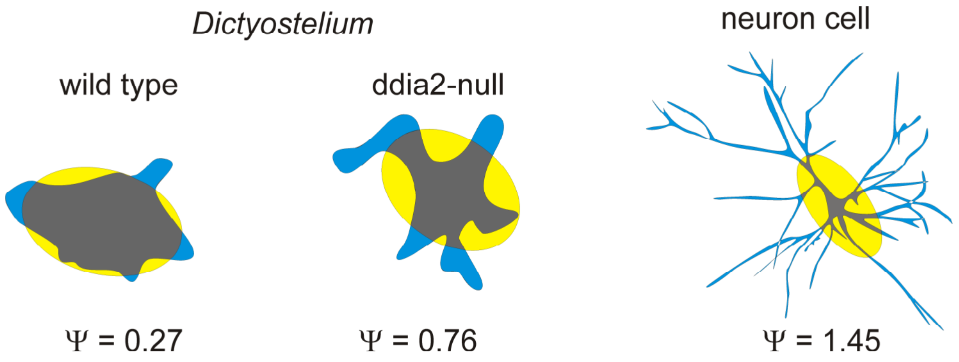

Figure S4. Determination of the shape parameter *Ψ*.
The cell outline is used to draw two ellipses, the largest possible ellipse inside the cell and the smallest possible ellipse outside the cell. Then an intermediate ellipse is constructed by interpolation of the inner and outer ellipse; the figure shows the intermediate ellipse. This ellipse intersects the outline, thereby forming the blue areas *O* of the cell that are outside the intermediate ellipse, and yellow areas *I* of the intermediate ellipsoid that do not belong to the cell. The intermediate ellipse was interpolated in such a way that $O = I$. The parameter of cell shape is defined as $\Psi = (O + I)/T$, where *T* is the surface area of the cell (grey + blue). The minimal value is $\Psi = 0$ when the cell is an ellipse, and the maximal value is $\Psi = 2$ for a cell with extreme long extensions; the maximal value observed among ~600 *Dictyostelium* cells is $\Psi = 0.92$. The neuron cell is shown for comparison.
